# Supplementary material for: Limitations and opportunities of smallholders’ practical knowledge when dealing with pig health issues in northern Uganda
Source: PLoS One. 2023 Jun 9;18(6):e0287041. doi: 10.1371/journal.pone.0287041 (PMC10256192; doi:10.1371/journal.pone.0287041)
Supplement: Attachment — Submitted filename: Response to reviewers.docx [file pone.0287041.s002.docx]

# Review PLOS One

We want to thank the two reviewers and the Editor for very helpful and constructive comments. Please find our responses to your comments below.

## Responses to reviewers' comments

**Reviewer 1:** A general comment: The authors should have crosschecked information with the veterinary authorities in Uganda.

**Response:** Thank you for your comment. We have now clarified that the information was crosschecked with veterinary authorities (including a District Veterinary Officer as well as veterinary officers working in northern Uganda) at the time of fieldwork in 2019. Please see lines 227-233 in attached “Revised Manuscript with Track Changes”.

**Reviewer 2:** L254: how recent is “more recently” maybe give a year or a range?

**Response:** We have provided more details on what “more recently” refers to in the given study context, please see lines 271-274.

**Reviewer 2:** L281: Would be good to mention the sex/gender for the quote and the name of the village in the whole text

**Response:** We have now stated the gender of the informants (please see revised manuscript). Due to promising the informants anonymity at the time of fieldwork in 2019, including not to specify the names of their villages, we have not been able to mention these in the manuscript however.

## Responses to Editor’s comments

**Editor:** Clarify the availability of veterinary services in the study area.

**Response:** Thank you for the comment. We have now provided more details on the availability of veterinary services in the study area, please see lines 382-385 and 389-391.

**Editor:** Clarify if the research team has triangulated some of the findings with local veterinary services

**Response:** We have now clarified the triangulation of findings with local veterinary services, please see lines 227-229.

**Editor:** More details on the farmers' experience rearing pigs can help to understand why they have poor knowledge about pig diseases and their management. For example, in neighboring countries (Kenya, Rwanda), pig rearing is perceived by smallholders as easier compared to other type of livestock production. There need to be more explanations on why the situation is different in the study area.

**Response:** Thanks for the helpful comment. We have now provided some additional details on smallholders’ experience of pig rearing, please see lines 271-274.

**Editor:** In the ethics statement in the Methods, you have specified that verbal consent was obtained. Please provide additional details regarding how this consent was documented and witnessed, and state whether this was approved by the IRB.

**Response:** Thank you for your comment. We have now clarified how the verbal consent was obtained, documented and witnessed. This was according to the IRB obtained prior to the fieldwork in Uganda. Please see lines 261-263.

**Editor:** We note that the grant information you provided in the ‘Funding Information’ and ‘Financial Disclosure’ sections do not match.

**Response:** Thank you for making us aware of this. We have now revised accordingly.

**Editor:** When you resubmit, please ensure that you provide the correct grant numbers for the awards you received for your study in the ‘Funding Information’ section.

**Response:** Thank you, we have made sure to provide the correct grant number in the ‘Funding Information’ section and provided an updated Funding Disclosure Statement in the attached Cover Letter.

**Editor:** In your Data Availability statement, you have not specified where the minimal data set underlying the results described in your manuscript can be found. PLOS defines a study's minimal data set as the underlying data used to reach the conclusions drawn in the manuscript and any additional data required to replicate the reported study findings in their entirety.

We will update your Data Availability statement to reflect the information you provide in your cover letter.

**Response:** The datasets cannot be shared publicly, as they contain both identifying and plausible sensitive participant information. The participants gave their consent with the understanding that their responses would remain confidential. With this said, researchers can request access to anonymized data by contacting the Swedish National Veterinary Institute (SVA) (contact via [sva@sva.se](mailto:sva@sva.se)). All other relevant data are available in the results section of the manuscript, including results from individual interviews and focus group discussions.

**Editor:** Your ethics statement should only appear in the Methods section of your manuscript. If your ethics statement is written in any section besides the Methods, please move it to the Methods section and delete it from any other section. Please ensure that your ethics statement is included in your manuscript, as the ethics statement entered into the online submission form will not be published alongside your manuscript.

**Response:** We have now double-checked to make sure that the ethics statement only appears in the Methods section.

**Editor:** Please review your reference list to ensure that it is complete and correct. If you have cited papers that have been retracted, please include the rationale for doing so in the manuscript text, or remove these references and replace them with relevant current references. Any changes to the reference list should be mentioned in the rebuttal letter that accompanies your revised manuscript. If you need to cite a retracted article, indicate the article’s retracted status in the References list and also include a citation and full reference for the retraction notice.

**Response:** We have now double-checked the references to ensure that no retracted papers are cited in the manuscript.

## Additional comment by the authors:

In addition to our responses to the reviewers’ and the editor’s comments outlined above, we also want to clarify that we have included a new reference (please see lines 165-166 and 182). This reference directs attention to our previous study among smallholder farmers in northern Uganda, in which descriptions of the study context as well as of the ethnographic fieldwork are provided. This reference was excluded upon our first submission to PLOS ONE, with respect to the blind review process. We have now included this reference with the aim of transparency, but have kept descriptions of the study villages as well as methodology and methods for readability purposes.
